# Clinical Features and Management of Lung Cancer During Pregnancy: A Narrative Review Based on Reported Cases

**DOI:** 10.1089/whr.2023.0085

**Published:** 2023-11-17

**Authors:** Jian Ping Zhou, Yi Wang, Ying Ni Lin, Xian Wen Sun, Yong Jie Ding, Ya Ru Yan, Ning Li, Liu Zhang, Qing Yun Li

**Affiliations:** ^1^ Department of Respiratory and Critical Care Medicine, Ruijin Hospital, Shanghai Jiao Tong University School of Medicine, Shanghai, China.; ^2^ Institute of Respiratory Diseases, Shanghai Jiao Tong University School of Medicine, Shanghai, China.

**Keywords:** pregnancy, lung cancer, diagnosis, treatment, outcomes

## Abstract

This review aims to provide a summary of the clinical characteristics and outcomes of lung cancer during pregnancy. A comprehensive literature search yielded 93 cases of lung cancer during pregnancy from 1953 to 2022, with an average maternal age of ∼34 years old. The initial symptoms reported were often nonspecific, such as cough, dyspnea, and chest pain. Cancer-related treatments, including surgery, radiotherapy, chemotherapy, and tyrosine kinase inhibitors, have shown beneficial effects on maternal outcomes. A majority of the newborns were born without malformation or diseases, but extended follow-up remains necessary. Early diagnosis of lung cancer is imperative for reducing the risks of placental and fetal metastasis and enhancing overall survival.

## Introduction

The occurrence of malignant tumors during pregnancy has garnered increasing attention due to its relatively higher prevalence and adverse impacts on both maternal and neonatal outcomes. The emerging ethical issues will also pose a significant challenge for clinical decision-making.^1^ While the incidence of lung cancer during pregnancy is lower compared to breast cancer, cervical cancer, and Hodgkin's lymphoma, it has been increasingly documented over the past two decades.^2^ The first case of lung cancer during pregnancy was reported by Barr,^3^ following by more than 90 subsequent cases.

Regrettably, nonspecific warning symptoms are often overlooked by pregnant women and health care professionals, leading to delayed diagnosis and eventual progression to advanced stages. An exploration of the clinical characteristics of lung cancer during pregnancy can provide valuable insights into risk factors, early detection, and personalized and appropriate treatment choices when in an ethical dilemma. Consequently, this review aims to synthesize findings from reported cases to summarize the manifestations and treatment approaches associated with lung cancer during pregnancy.

## Materials and Methods

**FIG. 1.**
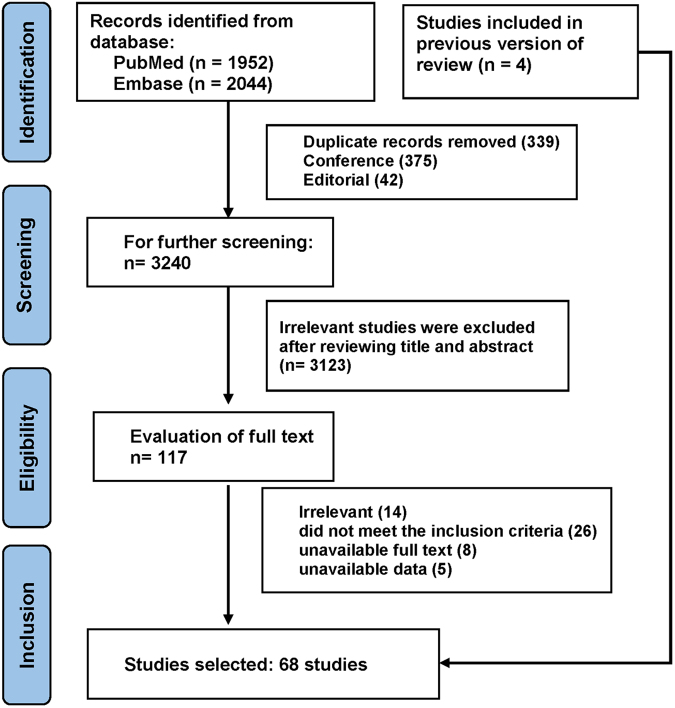
Literature search process.

We conducted a comprehensive literature search following the PRISMA guidelines,^4^ utilizing the PubMed and Embase databases. The mesh keywords employed included “pregnancy,” “lung cancer,” and “lung neoplasms” without any language restrictions. We individually reviewed publications that reported one or more cases of lung carcinoma coinciding with pregnancy. Our inclusion and exclusion criteria were as follows: (1) inclusion of patients who were pregnant; (2) inclusion of patients diagnosed with primary lung cancer; (3) requirement for full-text manuscripts and the availability of quantitative data; (4) exclusion of studies reporting that the same subjects were excluded from the analysis. Two investigators meticulously reviewed the titles, abstracts, and full texts of the selected studies. In instances of uncertain literature, discussions were conducted with another author to reach a final decision (Fig. 1).

Histopathological diagnosis encompassed small cell lung cancer (SCLC) and nonsmall cell lung cancer (NSCLC), including adenocarcinoma (ADC), squamous cell carcinoma (SCC), large cell carcinoma (LCC), and cases of unidentified NSCLC. The pregnancy period was categorized into three trimesters: the first trimester (≤12 weeks), the second trimester (>12 weeks, ≤28 weeks), and the third trimester (>28 weeks). Delivery outcomes were classified as extremely preterm delivery (before 28 weeks of gestation), preterm delivery (28–37 weeks of gestation), and full-term delivery. Cancer-related treatment (CRT) included surgery, chemotherapy, radiotherapy, and targeted therapy. Maternal outcomes were assessed using median survival (MS), calculated by Kaplan–Meier method in SPSS (version 19.0, IL, USA). Quantitative data were presented as median (range).

## Results

Over the past 69 years (1953–2022), a total of 93 pregnant women with lung cancer were identified in the databases (Supplementary Table S1). The incidence of reported cases of lung cancer has shown an upward trend over the last two decades, with 82.8% of cases (77 out of 93) reported since the year 2000.

### Demographic characteristics and diagnosis

A total of 93 patients were included in the analysis, with a median age of 34 years (ranging from 17 to 47 years). The median gestational week at the time of diagnosis was the 26th week. The most common symptoms were the “CDP” triad, which included cough (presented in 41.9% of cases), dyspnea (34.4%), and chest pain (15.1%). Additionally, symptoms related to metastatic sites were reported in 35.5% of cases. Among the 71 cases with documented smoking history, 26 (36.6%) were either current smokers or had a history of smoking (Table 1). Notably, 12 pregnant patients received empirical antibiotic therapy due to suspected pulmonary infections.

**Table 1.** Demographic Characteristics

No. of cases published	93
Median age (range)	34 (17–47)
Diagnosis time	
Before pregnancy	8
During pregnancy	78^a^
Post delivery	7
Median delivery week	33 (25–42)
Histopathology, *n* (%)	
SCLC	12 (12.9)
NSCLC	81 (87.1)
ADC	56 (60.2)
SCC	10 (10.8)
LCLC	8 (8.6)
Others	7 (7.5)
Smoking history, *n* (%)	
Smoker	26 (28.0)
Nonsmoker	45 (48.4)
Unknown	22 (23.7)
TNM stage, *n* (%)	
Ⅰ–Ⅱ	3 (3.2)
Ⅲ	8 (8.6)
Ⅳ	78 (83.9)
Unknown	4 (4.3)

^a^ The median gestational week when the diagnosis was confirmed was 26th week.

ADC, adenocarcinoma; NSCLC, non-small cell lung cancer; SCC, squamous cell carcinoma; SCLC, small cell lung cancer.

### Tumor typing and staging

Among the cases reviewed, 81 were classified as NSCLC, with further subtypes identified: ADC in 56 cases, SCC in 10 cases, LCC in 8 cases, and other subtypes in 7 cases. Additionally, there were 12 cases of SCLC. A majority of cases, comprising 86.0%, were diagnosed at an advanced stage, specifically at TNM staging IIIB–IV.

### Diagnostic procedures

During pregnancy, 44 patients (46.8%) underwent diagnostic procedures involving radiation exposure, including X-ray and computed tomography (CT). The diagnosis of lung cancer was subsequently confirmed through various methods, including lung biopsy (*n* = 39) which involved procedures such as bronchoscopy and CT-guided percutaneous biopsy, lymph node biopsy (*n* = 10), pleural effusion testing (*n* = 5), autopsy (*n* = 5), and surgical interventions (*n* = 5). Notably, nine cases were found to have epidermal growth factor receptor (EGFR)-positive ADC, and 15 cases were anaplastic lymphoma kinase (ALK)-positive ADC, confirmed through immunohistochemistry or genetic testing. The first documented EGFR mutation case was in 2007.^5^

### Placental metastases

Placental metastases were detected in 35.6% of the 45 cases that underwent histological examination of the placenta. Importantly, in some cases, metastases were observed in newborns, including one case with metastases on the head scalp and another with liver and lung metastases, which were identified at 2 weeks and 5 months of age, respectively.^6^

### Treatment strategy and maternal/neonatal outcomes

Intrapartum CRT was administered to 34 (36.6%) patients, distributed across the first (*n* = 5), the second (*n* = 23), and the third (*n* = 6) trimesters. Notably, four patients inadvertently received chemotherapy ([docetaxel, gemcitabine, and cisplatin],^7^ EGFR-tyrosine kinase inhibitor [TKI] [erlotinib],^8,9^ or ALK-TKI [alectinib]^10^) during the first trimester because of unrecognized conception, and their babies showed no evidence of congenital malformations. An additional 44 patients started CRT after delivering or terminating their pregnancy, while 12 patients did not receive any treatment. Cesarean sections were performed for 52 patients, while 15 opted for natural labor. Twelve patients chose pregnancy termination.

In terms of maternal outcomes, 8 (8.6%) patients died within 1 month after diagnosis. Twenty-five cases (26.9%) survived for 1–6 months, 23 cases (24.7%) survived for 6–12 months, and 32 cases (33.3%) survived for more than 12 months. The postdiagnosis MS was 11 months of all patients and 17 months for those who received CRT, respectively. Patients who underwent targeted therapy following positive EGFR or ALK detection had a higher MS, exceeding 24 months.

A total of 83 newborns were delivered, including 5 extremely preterm, 54 preterm, and 16 full-term births. The median gestational age was 33 weeks (ranging from 25 to 42 weeks). Most neonates showed no malformations or diseases during a median follow-up of 1.5 months (ranging from 0 to 38 months). However, two newborns were reported to have other diseases: familial hexadactyly (born in the 36th week)^11^ and necrotizing enterocolitis (born in the 26th week).^12^ Two newborns were found to have metastases, with one recovering from lung cancer metastases,^13^ and the other succumbing to extensive metastases.^14^ Additionally, Dagogo-Jack et al. reported that one extremely preterm infant (born in the 25th week) died 3 weeks after birth^15^ (Tables 2 and 3).

**Table 2.** Treatment and Outcome of Pregnant Patients with Lung Cancer

Treatment timing, *n* (%)	
Intrapartum	34 (36.6)
Postpartum/postabortion	44 (47.3)
No treatment	12 (12.9)
Unknown	3 (3.2)
Treatment choice, *n* (%)	
Radiotherapy	30 (32.3)
Chemotherapy	46 (49.5)
EGFR-TKI	14 (15.1)
ALK-TKI	15 (16.1)
Maternal outcome, *n* (%)	
Death in 1 month postpartum	8 (8.6)
Alive in 1–3 months	15 (16.1)
Alive in 3–6 months	10 (10.8)
Alive in 6–12 months	23 (24.7)
Alive in 12 month and more	31 (33.3)
Unknown	6 (6.5)
Placental metastases, *n* (%)	
Yes	16 (17.2)
No	29 (31.2)
Unknown	48 (51.6)
Fetal outcome, *n* (%)	
No evidence of disease	70 (84.3)
Fetal metastases	2 (2.4)
Other diseases or death	3 (3.6)^a^
Unknown	8 (9.6)

^a^ Including one with familial hexadactyly, one with necrotizing enterocolitis, and one death.

ALK, anaplastic lymphoma kinase; EGFR, epidermal growth factor receptor; TKI, tyrosine kinase inhibitors; NA, not available; NED, no evidence of disease.

**Table 3.** Maternal and Fetal Outcome after Receiving Intrapartum Cancer-Related Therapy

	Radiotherapy	Chemotherapy	TKIs	Radiosurgery	Surgery	Trastuzumab
Number	8	14	12	3	4	1
Maternal outcome	Death (4); Alive (4)	Death (9); Alive (5)	Death (3); Alive (9)	Alive (2); Death (1)	Alive (4)	Alive (1)
Fetal outcome	NED (8)	NED (13); necrotizing enterocolitis (1);	NED (12)	Abortion (1); NED (2)	NED (4)	NED (1)

NED (at the delivery or longer follow-up).

NED, no evidence of disease.

## Discussion

Our study revealed that lung cancer cases diagnosed during pregnancy often presented in advanced stage due to nonspecific symptoms. Risk factors included a delay in maternal age (around 34 years) and a history of smoking. Placental and fetal metastases were observed in some cases. The MS of the 93 cases analyzed was 11 months. With the advantage of precision medicine in the 21st century, cases with EGFR mutations or ALK rearrangements have benefited from targeted treatments, surpassing the effectiveness of traditional CRT strategy.

The association between lung cancer and pregnancy has gained increasing significance over the past two decades, representing an important yet complex issue that requires consideration for both the expectant mother and the newborn. A delay in maternal age and a history of smoking were identified as potential risk factors.^16^

Herein, we found that pregnant patients with lung cancer was typically in their thirties. The trend of women having fewer pregnancies at later ages, combined with the age-related increase in the incidence of most malignancies, may contribute to the rising incidence of pregnancy-associated cancer.^17,18^ A smoking history was identified in more than one-third of pregnant patients. Women are more susceptible to cigarette-induced carcinogenesis, which results from a lower DNA repair capacity.^19^ Therefore, delayed pregnancy and smoking can increase the risk of lung cancer. Furthermore, it has been reported that a longer fertility span and later menopause are associated with lung ADC, though the potential fluctuations during pregnancy and lung cancer risk remain unexplored.^20^

Delayed diagnosis often leads to a poor prognosis in pregnant women, who are more likely to be diagnosed with advanced lung cancer compared to the general population.^21^ Several factors contribute to this delay. First, patients frequently attribute early symptoms such as cough, dyspnea, chest pain, and fatigue to pregnancy-associated discomfort, leading them to disregard these symptoms. For example, some mothers may postpone seeking treatment for respiratory discomfort and progressive visual loss until after childbirth.^22^ Second, clinicians may not readily consider lung cancer diagnosis in pregnant women in their thirties, as pulmonary malignancies are more commonly associated with individuals in their fifth, sixth, or seventh decades of life. Third, symptoms often mimic those of benign pulmonary conditions such as pneumonia and asthma, which can result in the prescription of empirical antibiotics and bronchodilators.^23,24^ Finally, physicians may hesitate to employ radiological assessment and invasive procedures due to concerns about radiation exposure and potential harm. Pregnant individuals are also typically apprehensive about radiation exposure.

In this aspect, our review highlights the importance of considering radiation assessment with fetal shielding and the judicious use of invasive diagnostic procedures in the pregnant women. Notably, Chest X-ray and CT assessments were conducted in nearly 50% of the patients during pregnancy in this review. For those requiring a pathological diagnosis, bronchoscopy, lung biopsy *via* bronchoscopy, CT-guided percutaneous puncture, and surgery were carried out in 68.08% of patients without additional complications. Consequently, increasing awareness of lung cancer among both patients and health care providers, utilizing low-dose CT with shielding, and considering comprehensive check-ups can contribute to earlier diagnoses.

During pregnancy, alterations in drug metabolism and elimination can result in distinct toxicity patterns, which may indirectly affect the fetus.^25^ General risks associated with chemotherapy include preterm delivery, low birth weight, transient tachypnea of the newborn, and transient neonatal leucopenia.^26–28^ Typically, it is advisable to withhold 3 weeks before delivery or after reaching 35 weeks of gestation to minimize the risks of sepsis and hemorrhage in both the mother and newborn.^27,29^ Chemotherapy can resume after a sufficient recovery following delivery.

Radiotherapy during pregnancy is usually limited to cases where the tumor is located outside the pelvic region.^30^ In the context of lung cancer, intrapartum radiation therapy is primarily used for life-saving purposes or to preserve organ function such as radiosurgery for brain and bone metastases.^9,31–35^ When radiotherapy is considered necessary during pregnancy, it's important to consider using fetal shielding or engage in discussions regarding elective early delivery.^36,37^

Patients prescribed with EGFR-TKI or ALK-TKI experienced longer survival compared to those receiving traditional chemoradiotherapy. Notably, 12 cases^7–11,35,38–41^ administered TKIs during pregnancy, with three cases initiating medication in the first trimester.^8–10^ Importantly, no malformation was identified in the newborns. However, the safety of TKIs in pregnant women remains a subject of investigation, highlighting the need for further research to bridge existing gaps regarding the use of antitumor drugs during pregnancy.^42^ This is particularly pertinent given the emergence of new promising therapeutic options, such as immunotherapy and targeted therapy.

With improved long-term survival rates for lung cancer patients,^43^ the question of whether and when female patients with a history of lung cancer can consider pregnancy after treatment has gained significance. In our study, eight patients with a history of lung cancer reported unplanned or planned pregnancies during or after cancer treatment, which introduced complexities in treatment decisions and management. Therefore, it is crucial to rule out the possibility of conception before initiating treatment for female lung cancer patients of childbearing age to prevent the inadvertent use of antitumor drugs during pregnancy.

Given the potential for lung cancer to metastasize to the placenta and even the fetus, it is advisable to recommend microscopic histopathological examination of the placenta and thorough follow-up for these infants. Moreover, questions regarding the potential toxic effects of intrapartum TKI use, especially during the first trimester, on newborn health necessitate long-term monitoring.

This study has certain limitations. First, due to the limited number and incomplete data of reported cases, the study is susceptible to information bias. Second, the prognosis in lung cancer patients depends on multiple factors, including patient’ s overall health, the stage and type of lung cancer, treatment choice and timing, as well as the decision to terminate pregnancy or not. These factors can introduce bias when assessing survival times.

## Conclusion

The incidence of lung cancer during pregnancy is increasing, possibly due to delayed pregnancy and smoking habits. Managing lung cancer in pregnant individuals presents significant challenges, including timely detection, treatment decision, and balancing maternal health with effective tumor management. This review provides valuable clinical insights for health care professionals facing the complex scenario of pregnant patients with lung cancer.

## Supplementary Material

Supplemental data

## Abbreviations Used

ADC: adenocarcinoma
ALK: anaplastic lymphoma kinase
CRT: cancer-related treatment
CT: computed tomography
EGFR: epidermal growth factor receptor
LCC: large cell carcinoma
MS: median survival
NSCLC: non-small cell lung cancer
SCC: squamous cell carcinoma
SCLC: small cell lung cancer
TKI: tyrosine kinase inhibitor

## References

[1] Alpuim Costa D, Nobre JG, de Almeida SB, et al. Cancer during pregnancy: How to handle the bioethical dilemmas?—A scoping review with paradigmatic cases-based analysis. Front Oncol 2020;10:598508; doi: 10.3389/fonc.2020.598508 33425755 PMC7787159

[2] Voulgaris E, Pentheroudakis G, Pavlidis N. Cancer and pregnancy: A comprehensive review. Surg Oncol 2011;20(4):e175–e185; doi: 10.1016/j.suronc.2011.06.002 21733678

[3] Barr JS. Placental metastases from a bronchial carcinoma. J Obstet Gynaecol Br Emp 1953;60(6):895–897; doi: 10.1111/j.1471-0528.1953.tb07292.x 13131128

[4] Page MJ, McKenzie JE, Bossuyt PM, et al. The PRISMA 2020 statement: An updated guideline for reporting systematic reviews. BMJ 2021;372:n71; doi: 10.1136/bmj.n71 33782057 PMC8005924

[5] But Hadzic J, Secerov A, Zwitter M, et al. Metastatic adenocarcinoma of the lung in a 27-year-old pregnant woman. J Thorac Oncol 2007;2(5):450–452; doi: 10.1097/01.Jto.0000268680.33238.01 17473662

[6] Cancer Case Reports. Chest 1997;112(3, Supplement):159S–161S; doi: 10.1378/chest.112.3_Supplement.159S

[7] Kim JH, Kim HS, Sung CW, et al. Docetaxel, gemcitabine, and cisplatin administered for non-small cell lung cancer during the first and second trimester of an unrecognized pregnancy. Lung Cancer 2008;59(2):270–273; doi: 10.1016/j.lungcan.2007.06.017 17688967

[8] Zambelli A, Prada GA, Fregoni V, et al. Erlotinib administration for advanced non-small cell lung cancer during the first 2 months of unrecognized pregnancy. Lung Cancer 2008;60(3):455–457; doi: 10.1016/j.lungcan.2007.10.025 18063195

[9] Rivas G, Llinás N, Bonilla C, et al. Use of erlotinib throughout pregnancy: A case-report of a patient with metastatic lung adenocarcinoma. Lung Cancer 2012;77(2):469–472; doi: 10.1016/j.lungcan.2012.03.026 22534670

[10] Scarfone G, Fumagalli M, Imbimbo M, et al. First case report of pregnancy on alectinib in a woman with metastatic ALK-rearranged lung cancer: A case report. J Thorac Oncol 2021;16(5):873–877; doi: 10.1016/j.jtho.2021.02.005 33795207

[11] Boudy AS, Grausz N, Selleret L, et al. Use of tyrosine kinase inhibitors during pregnancy for oncogenic-driven advanced non-small cell lung carcinoma. Lung Cancer 2021;161:68–75; doi: 10.1016/j.lungcan.2021.09.001 34543940

[12] Boussios S, Han SN, Fruscio R, et al. Lung cancer in pregnancy: Report of nine cases from an International Collaborative Study. Lung Cancer 2013;82(3):499–505; doi: 10.1016/j.lungcan.2013.09.002 24091171

[13] Walker JW, Reinisch JF, Monforte HL. Maternal pulmonary adenocarcinoma metastatic to the fetus: First recorded case report and literature review. Pediatr Pathol Mol Med 2002;21(1):57–69; doi: 10.1080/pdp.21.1.57.69 11858176

[14] Teksam M, McKinney A, Short J, et al. Intracranial metastasis via transplacental (vertical) transmission of maternal small cell lung cancer to fetus: CT and MRI findings. Acta Radiol 2004;45(5):577–579; doi: 10.1080/02841850410005660 15515522

[15] Dagogo-Jack I, Gainor JF, Porter RL, et al. Clinicopathologic features of NSCLC diagnosed during pregnancy or the peripartum period in the era of molecular genotyping. J Thorac Oncol 2016;11(9):1522–1528; doi: 10.1016/j.jtho.2016.05.031 27296107 PMC5002360

[16] Maggen C, Wolters V, Cardonick E, et al. Pregnancy and cancer: The INCIP Project. Curr Oncol Rep 2020;22(2):17; doi: 10.1007/s11912-020-0862-7 32025953 PMC7002463

[17] Whiteman DC, Wilson LF. The fractions of cancer attributable to modifiable factors: A global review. Cancer Epidemiol 2016;44:203–221; doi: 10.1016/j.canep.2016.06.013 27460784

[18] Troisi R, Bjørge T, Gissler M, et al. The role of pregnancy, perinatal factors and hormones in maternal cancer risk: A review of the evidence. J Intern Med 2018;283(5):430–445; doi: 10.1111/joim.12747 29476569 PMC6688839

[19] Henschke CI, Yip R, Miettinen OS. Women's susceptibility to tobacco carcinogens and survival after diagnosis of lung cancer. JAMA 2006;296(2):180–184; doi: 10.1001/jama.296.2.180 16835423

[20] Wilunda C, Sawada N, Yamaji T, et al. Reproductive factors and lung cancer risk among never-smoking Japanese women with 21 years of follow-up: A Cohort Study. Cancer Epidemiol Biomarkers Prev 2021;30(6):1185–1192; doi: 10.1158/1055-9965.Epi-20-1399 33827981

[21] Navani N, Baldwin DR, Edwards JG, et al. Lung cancer in the United Kingdom. J Thorac Oncol 2022;17(2):186–193; doi: 10.1016/j.jtho.2021.11.002 35074225

[22] Lin L, Sun J, Wang J. Lung cancer and intraocular metastasis in gestation: Clinical experiences of a rare case. Thorac Cancer 2020;11(9):2723–2726; doi: 10.1111/1759-7714.13572 32691515 PMC7471047

[23] Crosby E. Clinical case discussion: Anesthesia for Cesarean section in a parturient with a large intrathoracic tumour. Can J Anaesth 2001;48(6):575–583; doi: 10.1007/bf03016835 11444453

[24] Jänne PA, Rodriguez-Thompson D, Metcalf DR, et al. Chemotherapy for a patient with advanced non-small-cell lung cancer during pregnancy: A case report and a review of chemotherapy treatment during pregnancy. Oncology 2001;61(3):175–183; doi: 10.1159/000055371 11574771

[25] Isoherranen N, Thummel KE. Drug metabolism and transport during pregnancy: How does drug disposition change during pregnancy and what are the mechanisms that cause such changes? Drug Metab Dispos 2013;41(2):256–262; doi: 10.1124/dmd.112.050245 23328895 PMC3558867

[26] Cardonick E, Iacobucci A. Use of chemotherapy during human pregnancy. Lancet Oncol 2004;5(5):283–291; doi: 10.1016/s1470-2045(04)01466-4 15120665

[27] Cordeiro CN, Gemignani ML. Gynecologic malignancies in pregnancy: Balancing Fetal risks with oncologic safety. Obstet Gynecol Surv 2017;72(3):184–193; doi: 10.1097/ogx.0000000000000407 28304416 PMC5358514

[28] Ji YI, Kim KT. Gynecologic malignancy in pregnancy. Obstet Gynecol Sci 2013;56(5):289–300; doi: 10.5468/ogs.2013.56.5.289 24328018 PMC3784125

[29] Silverstein J, Post AL, Chien AJ, et al. Multidisciplinary management of cancer during pregnancy. JCO Oncol Pract 2020;16(9):545–557; doi: 10.1200/op.20.00077 32910882

[30] Mazonakis M, Damilakis J. Estimation and reduction of the radiation dose to the fetus from external-beam radiotherapy. Phys Med 2017;43:148–152; doi: 10.1016/j.ejmp.2017.09.130 28943130

[31] Mujaibel K, Benjamin A, Delisle MF, et al. Lung cancer in pregnancy: Case reports and review of the literature. J Matern Fetal Med 2001;10(6):426–432; doi: 10.1080/714052775 11798456

[32] Magné N, Marcié S, Pignol JP, et al. Radiotherapy for a solitary brain metastasis during pregnancy: A method for reducing fetal dose. Br J Radiol 2001;74(883):638–641; doi: 10.1259/bjr.74.883.740638 11509400

[33] Van Winter JT, Wilkowske MA, Shaw EG, et al. Lung cancer complicating pregnancy: Case report and review of literature. Mayo Clinic Proc 1995;70(4):384–387; doi: 10.1016/s0025-6196(11)63421-6 7898147

[34] Holzmann K, Kropfmüller R, Schinko H, et al. Lung cancer in pregnancy. Wien Klin Wochenschr 2015;127(15–16):639–644; doi: 10.1007/s00508-015-0726-6 25732916

[35] Ji Y, Schwartz J, Hartford A, et al. Successful treatment of non-small cell lung cancer with erlotinib throughout pregnancy. JAMA Oncol 2015;1(6):838–840; doi: 10.1001/jamaoncol.2015.1300 26181671

[36] Azim HA Jr, Scarfone G, Peccatori FA. Carboplatin and weekly paclitaxel for the treatment of advanced non-small cell lung cancer (NSCLC) during pregnancy. J Thorac Oncol 2009;4(4):559–560; doi: 10.1097/JTO.0b013e31819c8674 19333076

[37] Mitrou S, Zarkavelis G, Fotopoulos G, et al. A mini review on pregnant mothers with cancer: A paradoxical coexistence. J Adv Res 2016;7(4):559–563; doi: 10.1016/j.jare.2016.01.004 27408757 PMC4921772

[38] Lee CH, Liam CK, Pang YK, et al. Successful pregnancy with epidermal growth factor receptor tyrosine kinase inhibitor treatment of metastatic lung adenocarcinoma presenting with respiratory failure. Lung Cancer 2011;74(2):349–351; doi: 10.1016/j.lungcan.2011.08.008 21920622

[39] Gil S, Goetgheluck J, Paci A, et al. Efficacy and safety of gefitinib during pregnancy: Case report and literature review. Lung Cancer 2014;85(3):481–484; doi: 10.1016/j.lungcan.2014.06.003 24997732

[40] Padrão E, Melo C, Fernandes G, et al. Lung cancer in pregnancy—Report of a case treated with crizotinib. Pulmonology 2018;24(3):205–207; doi: 10.1016/j.pulmoe.2018.03.007 29754722

[41] Jensen KH, Persson G, Storgaard L, et al. Antineoplastic treatment with crizotinib during pregnancy: A case report. Acta Oncol 2019;58(1):121–122; doi: 10.1080/0284186x.2018.1497302 30101631

[42] Couzin-Frankel J. The pregnancy gap. Science 2022;375(6586):1216–1220; doi: 10.1126/science.adb2029 35298273

[43] Siegel RL, Miller KD, Fuchs HE, et al. Cancer statistics, 2022. CA Cancer J Clin 2022;72(1):7–33; doi: 10.3322/caac.21708 35020204

