# Supplementary material for: Clinical Features and Management of Lung Cancer During Pregnancy: A Narrative Review Based on Reported Cases
Source: Womens Health Rep (New Rochelle). 2023 Nov 17;4(1):544–50. doi: 10.1089/whr.2023.0085 (PMC10664570; doi:10.1089/whr.2023.0085)
Supplement: Supplemental data [file Suppl_TableS1.pdf]

Supplementary Table 1 Clinical data on all reported cases of lung cancer during pregnancy

Author	Year	Age	X-ray Exposure	Diagnosis method	Smoking status	Diagnosis	TNM	Gestational week at diagnosis	delivery week	Treatment and timing of treatment	maternal outcome (after diagnosis)	Placenta Invasion	Fetus outcome
Barr [1]	1953	39	YES	autopsy	NA	SCLC	IVB	22	32	NO	Death, 4 months	YES	NED at 3 years
Jones [2]	1969	39	NA	bronchoscopy	NO	SCLC	IVB	34	38	postpartum radiotherapy	Death, 9 months	YES	NED at 6 months
Pawelec [3]	1976	39	NA	NA	NA	ADC	IV	NA	NA	NA	NA	NA	NA
Read [4]	1981	37	NO	nodule biopsy	YES	LCLC	IVB	23	36	postpartum chemoradiotherapy	Death, 2 months	YES	NED at 16 months
Reiter [5]	1985	35	YES	bronchoscopy	NA	ADC	IVB	23	30	intrapartum radiotherapy and postpartum chemotherapy	Death, 2.3 month	NO	NED at 7 weeks
Stark [6]	1985	45	NA	NA	YES	SCLC	III	36	NA	postpartum chemotherapy	No follow-up	YES	NED at delivery
		34	NA	NA	YES	SCC	NA	40	NA	postpartum surgery	Death, 42 months	YES	NED at delivery
Suda [7]	1986	33	NA	bronchoscopy/autopsy	NO	LCLC	IV	22	NA	NO	Death; 2.5 months	YES	NED at delivery
Brühwiler [8]	1988	41	YES	biopsy	YES	LCLC	IV	37	37	postpartum chemoradiotherapy	Death; 3.5 months	NO	NED at delivery
Delerive [9]	1989	30	NA	lung biopsy	YES	SCLC	IV	32	32	postpartum chemotherapy	Alive; 6 months	YES	NED at 18 months
Dildy [10]	1989	44	NA	lung biopsy	NO	LCLC	IVB	prepregnant	35	postpartum radiotherapy	Death, 7 months	YES	NED at 40 days
Bitar [11]	1995	43	YES	bronchoscopy	YES	ADC	IVB	37	37	NO	Death, 1 months	NO	NED at 1 months
Cone [12]	1995	29	abortion	lung biopsy	NO	ADC	IVA	3rd month	abortion	post-abortion chemotherapy	Death, 62 months	NA	Abortion
Van Winter [13]	1995	36	YES	femur biopsy	YES	ADC	IVA	29	32	intrapartum radiotherapy on the thigh	Death, 8 months	NA	NED at 7 months
Abul-Khoudoud [14]	1997	17	YES	pleural effusion test	NO	SCC	IVA	27	29	postpartum chemotherapy	No follow-up	NO	NA
Chen [15]	1998	34	NA	NA	NO	ADC	IVA	36	NA	postpartum radiotherapy	Alive; 15 months	NA	NA
		31	NA	lung biopsy	NO	SCC	IVB	40	NA	postpartum chemoradiotherapy	Death, 2 months	NA	NA
Crosby [16]	2001	28	YES	lung biopsy	YES	SCLC	IVA	33	33	NA	NA	NO	NED at delivery
Jänne [17]	2001	31	YES	bronchoscopy	YES	ADC	IVA	26	26	intrapartum chemotherapy	Alive, 9 months	NO	NED at 9 months
Kochman [18]	2001	44	NA	lung biopsy	NA	SCLC	IVB	30	30	NO	Death, 16 days	YES	NED at delivery
Magné[19]	2001	38	NO	bronchoscopy	NA	ADC	IVA	prepregnant	40	intrapartum brain radiotherapy	Alive; 41 months	NA	NED at 38 months
Mujaibel [20]	2001	40	YES	bronchoscopy	YES	SCC	IIIA	29	34	postpartum radiotherapy then surgery	Alive, 13 months	NO	NED at 12 months
		35	YES	lung biopsy	YES	ADC	IVB	31	34	intrapartum brain radiotherapy	Death, 2.5 months	NO	NED at 2 weeks
		28	YES	bronchoscopy	NA	NSCLC	IVA	postpartum	29	postpartum chemotherapy	Alive, 12 months	NA	NED at 4 weeks

Walker [21]	2002	46	YES	lymph node biopsy	NO	ADC	IVB	23	32	NO	Death, 2 days	NA	scalp nodules found at 2 weeks of age. Free of disease at five years old
Jackisch [22]	2003	29	YES	lung biopsy	NO	LCLC	IVB	31	33	NO	Death, 1 months	YES	NED at 15 months
Wong [23]	2003	30	YES	bronchoscopy	NO	SCC	IVB	31	35	NO	Death, 8 months	NA	NED at delivery
		33	NO	lymph node biopsy	YES	ADC	IVB	35	35	postpartum chemotherapy	Death, 5 months	NA	NED at delivery
Teksam [24]	2004	37	YES	lung biopsy	YES	SCLC	IVB	33	33	postpartum chemotherapy	Death, 5 months	YES	Lung, liver, brain metastases and died
Adams [25]	2004	35	NO	postpartum bronchoscopy	NO	ADC	IVB	31	31	postpartum brain radiation, chemotherapy	Death, 18 weeks	NA	NED at delivery
Folk [26]	2004	41	NA	lymph node biopsy	NA	ADC	IVB	35	35	NO	Death, a few weeks	YES	NED at 6 months
Penha [27]	2006	25	NO	autopsy	YES	SCC	IVB	postpartum	37	NO	Death, 10 days	NA	NED at delivery
Innamaa [28]	2006	34	YES	bronchoscopy	NO	ADC	IVB	31	31	postpartum brain radiation and chemotherapy	Death, 4 months	YES	NED at 4 months
Burlacu [29]	2007	33	YES	lymph node biopsy	YES	ADC	IVB	16	30	postpartum chemoradiotherapy	Alive, 20.5 months	NO	NED at 18 months
But Hadzic [30]	2007	27	YES	lymph node biopsy; autopsy	NO	ADC	IVA	24	29	NO	Death, 5 weeks	NO	NED at 10 weeks
García-González [31]	2008	39	YES	lymph node biopsy	YES	ADC	IVB	17	30	intrapartum chemotherapy	Death, 10 months	NA	NED at 15 months
Garrido [32]	2008	34	YES	lung biopsy and bronchoscopy	NO	ADC	IIIB	27	39	intrapartum chemotherapy and postpartum brain radiotherapy	Alive, 16 months	NA	NED at 16 months
Kim [33]	2008	35	YES	brain surgery	NO	ADC	IVA	prepregnant	33	intrapartum brain radiotherapy then chemotherapy then EGFR-TKI	Alive, 10 months	NO	NED at 2.5 months
Kluetz [34]	2008	39	YES	lung biopsy	YES	SCLC	III	26	34	intrapartum chemotherapy and postpartum chemoradiotherapy	Death, 12 months	NO	NED at delivery
Zambelli [35]	2008	30	NO	bronchoscopy	NA	ADC	IVB	prepregnant	42	intrapartum EGFR-TKI	Alive, 12 months	NA	NED at 1 months
Azim [36]	2009	33	NO	biopsy	NO	ADC	IVA	19	30	intrapartum chemotherapy	Death, 15 weeks	NA	NED at 5 months
Gurumurthy [37]	2009	38	YES	bronchoscopy	NO	SCC	IVB	25	28	intrapartum chemotherapy	Death, 5 weeks	NO	NED at 8 months
Hata [38]	2009	34	YES	lymph node biopsy	NA	ADC	IVA	33	33	postpartum EGFR-TKI then chemotherapy	Death, 17 days	NA	NA
Thelmo [39]	2010	31	YES	pleural effusion test	NO	ADC	IVB	10	34	NO	Death, 7 months	NA	NED at delivery
Liu [40]	2010	31	YES	postpartum lymph-	NO	LCLC	IVA	35	35	postpartum EGFR-TKI	Death, 4 weeks	NA	NED at 12 months

				node biopsy									
	2010	28	abortion	postpartum bronchoscopy	NO	SCLC	IVB	27	abortion	post-abortion chemotherapy	Alive, 6 weeks	NA	Abortion
Lee [41]	2011	38	YES	Lung biopsy	NO	ADC	IVA	26	36	intrapartum EGFR-TKI	Alive, 16 weeks	NA	NED at 6 weeks
Rivas [42]	2012	40	Possible	before-pregnancy bronchoscopy	NO	ADC	IVB	prepregnant	33	intrapartum brain radiotherapy and EGFR-TKI	Alive, 11 months	NO	NED at 4 months
Boussios [43]	2013	33	YES	bronchoscopy	NA	ADC	IVB	28	30	postpartum chemoradiotherapy	Death, 10.5 months	NA	NED at delivery
		42	abortion	NA	NA	NSCLC	IVB	9	abortion	post-abortion radiotherapy and EGFR-TKI	Death, 2 months	NO	Abortion
		26	NO	NA	NO	NSCLC	IVB	17	abortion	intrapartum brain radiotherapy and chemotherapy	Death, 12 months	NA	Abortion
		25	NO	NA	NO	SCC	IVA	19	abortion	post-abortion chemotherapy	Death, 1 months	NA	Abortion
		31	YES	bronchoscopy	NA	LCLC	IVA	19	33rd	intrapartum chemotherapy	Death, 14 weeks	NO	NED at delivery
		32	NA	NA	NA	NSCLC	IVA	12	abortion	NA	NA	NA	Abortion
		34	NA	lung surgery	NA	ADC	T3	6	33	intrapartum chemotherapy	Death, 28 weeks	NA	NED at delivery
		42	NA	NA	YES	ADC	IVB	13	27	intrapartum chemotherapy	Death, 14 weeks	NA	NED at delivery
		30	NA	lung biopsy	YES	ADC	IVB	20	26	intrapartum chemotherapy	Death, 9 weeks	NA	necrotizing enterocolitis at 4 weeks
Sarıman [44]	2013	34	NO	postpartum bronchoscopy/lymph node biopsy	YES	ADC	IVB	postpartum	28	postpartum chemotherapy ALK-TKI	Alive, 6 months	NA	NED at 6 weeks
Gil [45]	2014	33	YES	bronchoscopy and lung biopsy	YES	ADC	IVA	26	35	intrapartum EGFR-TKI	Death, 22 months	NO	NED at 24 months
Hayama [46]	2014	34	YES	bronchoscopy	NO	ADC	IIIB	30	32	postpartum surgery and chemotherapy	Death, 8 months	NO	NED at delivery
Kim [47]	2014	38	YES	lung surgery	NO	LCLC	IIB	24	37	intrapartum surgery	Alive, 10 months	NO	NED at 6 months
Neves [48]	2014	36	NO	lung biopsy	YES	ADC	IVB	27	29	postpartum chemoradiotherapy and ALK-TKI	Death, 19 months	NA	NED at 47 days
Ceauşu [49]	2014	34	YES	bronchoscopy	NO	ADC	IVB	30	30	NO	Death, 1 week	NO	NED at delivery
Holzmann [50]	2015	29	YES	bronchoscopy	YES	ADC	IVB	26	31	intrapartum spine radiotherapy, postpartum EGFR-TKI and chemotherapy	Death, 17 months	NA	NED at delivery
Ji [51]	2015	47	NA	NA	NO	ADC	IVB	10	37	intrapartum brain radiotherapy+EGFR-TKI	Alive, 19.5 months	NO	NED at 12 months
Yates [52]	2015	26	YES	bronchoscopy	NO	SCC	IIIA	18	35	intrapartum chemotherapy and postpartum radiotherapy	Alive, 16 months	NA	NED at delivery

Cherian [53]	2016	34	YES	bronchoscopy	YES	ADC	T4	30	NA	postpartum chemotherapy	Death, 2 years	NA	NA
Dagogo-Jack [54]	2016	36	NA	NA	NO	ADC	IVA	30	32	postpartum ALK-TKI	Alive, 5 years	NA	NED at delivery
		36	NA	NA	YES	ADC	IVB	postpartum	NA	postpartum ALK-TKI	Death, 2 years	NA	NED at delivery
		33	NA	NA	NO	ADC	IVB	5	abortion	post-abortion ALK-TKI	Alive, 2.5 years	NA	Abortion
		29	NA	NA	NO	ADC	IVB	9	34	intrapartum chemotherapy and brain radiotherapy, postpartum ALK-TKI	Alive, 3 years	NA	NED at delivery
		35	NA	NA	NO	ADC	IVB	15	abortion	post-abortion ALK-TKI	Alive, 2.5 years	NA	Abortion
		31	NA	NA	YES	ADC	IVB	28	29	postpartum ALK-TKI	Alive, 6 months	NA	NED at delivery
		43	NA	NA	NO	ADC	IVA	postpartum	NA	postpartum EGFR-TKI	Alive, 5+ years	NA	NED at delivery
		35	NA	NA	NO	ADC	IVB	postpartum	25	postpartum EGFR-TKI	Alive, 11 months	NA	Died three weeks after delivery
Safini [55]	2016	23	NA	NA	NO	SCLC	IIIA	10	37	intrapartum chemotherapy and postpartum chemoradiotherapy	Death, 13 months	NO	NED at delivery
Gziri [56]	2017	30	YES	bronchoscopy	NO	NSCLC	IIA	15	NA	intrapartum surgery	Alive, 7 months	NO	NED at delivery
Moro [57]	2017	24	abortion	post-abortion ovary surgery	NA	SCLC	IVB	6	abortion	post-abortion chemotherapy and immunotherapy	Alive, 14 months	NA	Abortion
Isci Bostanci [58]	2018	34	abortion	NA	NA	NSCLC	NA	prepregnant	abortion	post-abortion chemoradiotherapy	Death, 29 months	NO	Abortion
Komura [59]	2018	28	YES	pleural effusion test	NA	ADC	IVA	34	37	postpartum ALK-TKI	Alive, 12 months	NO	NED at 12 months
Padrão [60]	2018	36	YES	pleural effusion test	NA	ADC	IVB	22	30	intrapartum ALK-TKI and postpartum chemotherapy	Death, 3 months	YES	NED at 29 months
Bellido [61]	2019	42	YES	lymph node biopsy	NA	ADC	IVB	30	31	postpartum ALK-TKI + chemotherapy	Alive, 12 months	YES	NED at 12 months
Jensen [62]	2019	32	YES	NA	NA	ADC	IVA	20	26	intrapartum ALK-TKI	Death, 6 weeks (Hellp syndrome)	YES	NED at 18 months
Siddiqui [63]	2019	19	NA	bronchoscopy	NA	SCC	IB	4	36	intrapartum lung resection	Alive, 26 months	NA	NED at delivery
Watanabe [64]	2019	38	NO	postpartum (pleural-effusion test)	NO	ADC	IVB	35	37	postpartum EGFR-TKI	Alive, NA	NO	NA
Han [65]	2020	26	YES	postpartum(tracheoscopy)	NO	SCLC	III	37	37	postpartum chemoradiotherapy	Alive, 19 months	NA	NED at delivery
		39	NA	pleural effusion test	NO	ADC	IV	20	abortion	post-abortion chemotherapy	Death, 4 months	NA	Abortion
		26	abortion	lymph node biopsy	NO	ADC	IVB	22	abortion	post-abortion chemoradiotherapy	Death, 7 months	NA	Abortion
Lin [66]	2020	31	NO	NA	NO	ADC	IVB	postpartum	NA	postpartum chemotherapy	Death, 12 months	NA	NA

Boudy [67]	2021	42	YES	bronchoscopy	NO	NSCLC	IVB	31	36	intrapartum ALK-TKI	Alive, 12 months	NO	NED at 11 months
		34	YES	brain surgery	NO	ADC	IVB	22	30	intrapartum EGFR-TKI +Trastuzumab	Alive, 10 months	NO	NED at 8 months
		31	NO	NA	NO	ADC	IVB	prepregnant	36	intrapartum ALK-TKI	Alive, 50 months	NA	NED at 30 months
Scarfone [68]	2021	31	NA	NA	NA	ADC	IV	prepregnant	35	intrapartum ALK-TKI	Alive, 32 months	NO	NED at 20 months

NSCLC, non-small cell lung cancer; SCLC, small cell lung cancer; ADC, adenocarcinoma; LCC, large cell carcinoma; SCC, squamous cell carcinoma; ALK, anaplastic lymphoma kinase; EGFR, epidermal growth factor receptor; TKI, tyrosine kinase inhibitors; NA, not available; NED, no evidence of disease; Hellp syndrome, hemolysis, elevated liver enzymes, and low platelets syndrome.

1. Barr JS. Placental metastases from a bronchial carcinoma. The Journal of obstetrics and gynaecology of the British Empire 1953;60(6):895-7, doi:10.1111/j.1471-0528.1953.tb07292.x
2. Jones EM. Placental metastases from bronchial carcinoma. British medical journal 1969;2(5655):491-2, doi:10.1136/bmj.2.5655.491
3. Pawelec D, Madey B. [Adenocarcinoma of the lung with metastasis in a 39-year-old pregnant woman]. Pneumonologia polska 1976;44(3):283
4. Read EJ, Jr., Platzer PB. Placental metastasis from maternal carcinoma of the lung. Obstetrics and gynecology 1981;58(3):387-91
5. Reiter AA, Carpenter RJ, Dudrick SJ, et al. Pregnancy associated with advanced adenocarcinoma of the lung. International journal of gynaecology and obstetrics: the official organ of the International Federation of Gynaecology and Obstetrics 1985;23(1):75-8, doi:10.1016/0020-7292(85)90017-7
6. Stark P, Greene RE, Morgan G, et al. Lung cancer and pregnancy. Der Radiologe 1985;25(1):30-2
7. Suda R, Repke JT, Steer R, et al. Metastatic adenocarcinoma of the lung complicating pregnancy. A case report. The Journal of reproductive medicine 1986;31(12):1113-6
8. Brühwiler H, Wild A, Lüscher KP. [Bronchus cancer and pregnancy]. Geburtshilfe und Frauenheilkunde 1988;48(9):654-5, doi:10.1055/s-2008-1026559
9. Delerive C, Locquet F, Mallart A, et al. Placental metastasis from maternal bronchial oat cell carcinoma. Archives of pathology & laboratory medicine 1989;113(5):556-8
10. Dildy GA, 3rd, Moise KJ, Jr., Carpenter RJ, Jr., et al. Maternal malignancy metastatic to the products of conception: a review. Obstetrical & gynecological survey 1989;44(7):535-40, doi:10.1097/00006254-198907000-00008
11. Bitar RJ, Melillo N, Pesin JL. Lung cancer during pregnancy. Mayo Clinic proceedings 1995;70(11):1130, doi:10.4065/70.11.1130
12. Cone LA, Dawson AC, Mata AM. Lung cancer during pregnancy. Mayo Clinic proceedings 1995;70(11):1130, doi:10.4065/70.11.1130-a
13. Van Winter JT, Wilkowske MA, Shaw EG, et al. Lung cancer complicating pregnancy: case report and review of literature. Mayo Clinic proceedings 1995;70(4):384-7, doi:10.1016/s0025-6196(11)63421-6
14. Abul-Khoudoud MR, Lwebuga-Mukasa JS. Primary Lung Cancer in A Seventeen Year Old, Twenty-seven-week Pregnant Woman. Chest 1997;112(3, Supplement):159S-161S, doi:https://doi.org/10.1378/chest.112.3_Supplement.159S
15. Chen KY, Wang HC, Shih JY, et al. Lung cancer in pregnancy: report of two cases. Journal of the Formosan Medical Association = Taiwan yi zhi 1998;97(8):573-6
16. Crosby E. Clinical case discussion: anesthesia for Cesarean section in a parturient with a large intrathoracic tumour. Canadian journal of anaesthesia = Journal canadien d'anesthesie 2001;48(6):575-83, doi:10.1007/bf03016835
17. Jänne PA, Rodriguez-Thompson D, Metcalf DR, et al. Chemotherapy for a patient with advanced non-small-cell lung cancer during pregnancy: a case report and a review of chemotherapy treatment during pregnancy. Oncology 2001;61(3):175-83, doi:10.1159/000055371
18. Kochman AT, Rabczyński JK, Baranowski W, et al. Metastases to the products of conception from a maternal bronchial carcinoma. A case report and review of literature. Polish journal of pathology : official journal of the Polish Society of Pathologists 2001;52(3):137-40
19. Magné N, Marcié S, Pignol JP, et al. Radiotherapy for a solitary brain metastasis during pregnancy: a method for reducing fetal dose. The British journal of radiology 2001;74(883):638-41, doi:10.1259/bjr.74.883.740638
20. Mujaibel K, Benjamin A, Delisle MF, et al. Lung cancer in pregnancy: case reports and review of the literature. The Journal of maternal-fetal medicine 2001;10(6):426-32, doi:10.1080/714052775
21. Walker JW, Reinisch JF, Monforte HL. Maternal pulmonary adenocarcinoma metastatic to the fetus: first recorded case report and literature review. Pediatric pathology & molecular medicine 2002;21(1):57-69, doi:10.1080/pdp.21.1.57.69
22. Jackisch C, Louwen F, Schwenkhagen A, et al. Lung cancer during pregnancy involving the products of conception and a review of the literature. Archives of gynecology and obstetrics 2003;268(2):69-77, doi:10.1007/s00404-002-0356-x
23. Wong CM, Lim KH, Liam CK. Metastatic lung cancer in pregnancy. Respirology (Carlton, Vic) 2003;8(1):107-9, doi:10.1046/j.1440-1843.2003.00423.x
24. Teksam M, McKinney A, Short J, et al. Intracranial metastasis via transplacental (vertical) transmission of maternal small cell lung cancer to fetus: CT and MRI findings. Acta radiologica (Stockholm, Sweden : 1987) 2004;45(5):577-9, doi:10.1080/02841850410005660
25. Adams FR, Levy DM, Reid MF, et al. All that glisters...a generalised seizure at 31 weeks. Journal of obstetrics and gynaecology : the journal of the Institute of Obstetrics and Gynaecology 2004;24(2):174-5, doi:10.1080/01443610410001648304
26. Folk JJ, Curioca J, Nosovitch JT, Jr., et al. Poorly differentiated large cell adenocarcinoma of the lung metastatic to the placenta: a case report. The Journal of reproductive medicine 2004;49(5):395-7
27. Penha DS, Salge AK, Tironi F, et al. Bronchogenic carcinoma of squamous cells in a young pregnant woman. Annals of diagnostic pathology 2006;10(4):235-8, doi:10.1016/j.anndiagpath.2005.09.014
28. Innamaa A, Deering P, Powell MC. Advanced lung cancer presenting with a generalized seizure in pregnancy. Acta obstetricia et gynecologica Scandinavica 2006;85(9):1148-9, doi:10.1080/00016340600604062
29. Burlacu CL, Fitzpatrick C, Carey M. Anaesthesia for caesarean section in a woman with lung cancer: case report and review. International journal of obstetric anesthesia 2007;16(1):50-62, doi:10.1016/j.ijoa.2006.04.014

30. But Hadzic J, Secerov A, Zwitter M, et al. Metastatic adenocarcinoma of the lung in a 27-year-old pregnant woman. Journal of thoracic oncology : official publication of the International Association for the Study of Lung Cancer 2007;2(5):450-2, doi:10.1097/01.Jto.0000268680.33238.01

31. García-González J, Cueva J, Lamas MJ, et al. Paclitaxel and cisplatin in the treatment of metastatic non-small-cell lung cancer during pregnancy. Clinical & translational oncology : official publication of the Federation of Spanish Oncology Societies and of the National Cancer Institute of Mexico 2008;10(6):375-6, doi:10.1007/s12094-008-0215-8

32. Garrido M, Clavero J, Huete A, et al. Prolonged survival of a woman with lung cancer diagnosed and treated with chemotherapy during pregnancy. Review of cases reported. Lung cancer (Amsterdam, Netherlands) 2008;60(2):285-90, doi:10.1016/j.lungcan.2007.09.019

33. Kim JH, Kim HS, Sung CW, et al. Docetaxel, gemcitabine, and cisplatin administered for non-small cell lung cancer during the first and second trimester of an unrecognized pregnancy. Lung cancer (Amsterdam, Netherlands) 2008;59(2):270-3, doi:10.1016/j.lungcan.2007.06.017

34. Kluetz PG, Edelman MJ. Successful treatment of small cell lung cancer during pregnancy. Lung cancer (Amsterdam, Netherlands) 2008;61(1):129-30, doi:10.1016/j.lungcan.2007.10.007

35. Zambelli A, Prada GA, Fregoni V, et al. Erlotinib administration for advanced non-small cell lung cancer during the first 2 months of unrecognized pregnancy. Lung cancer (Amsterdam, Netherlands) 2008;60(3):455-7, doi:10.1016/j.lungcan.2007.10.025

36. Azim HA, Jr., Scarfone G, Peccatori FA. Carboplatin and weekly paclitaxel for the treatment of advanced non-small cell lung cancer (NSCLC) during pregnancy. Journal of thoracic oncology : official publication of the International Association for the Study of Lung Cancer 2009;4(4):559-60, doi:10.1097/JTO.0b013e31819c8674

37. Gurumurthy M, Koh P, Singh R, et al. Metastatic non-small-cell lung cancer and the use of gemcitabine during pregnancy. Journal of perinatology : official journal of the California Perinatal Association 2009;29(1):63-5, doi:10.1038/jp.2008.128

38. Hata A, Harada Y, Seo R, et al. [A case of lung cancer combined with pregnancy; dramatically deteriorating condition after caesarian section]. Nihon Kokyuki Gakkai zasshi = the journal of the Japanese Respiratory Society 2009;47(7):585-90

39. Thelmo MC, Shen EP, Shertukde S. Metastatic pulmonary adenocarcinoma to placenta and pleural fluid: clinicopathologic findings. Fetal and pediatric pathology 2010;29(1):45-56, doi:10.3109/15513810903266625

40. Liu LY, Zhang WZ, Guan J, et al. [Lung cancer in pregnancy: report of two cases and review of literature]. Zhonghua jie he he hu xi za zhi = Zhonghua jiehe he huxi zazhi = Chinese journal of tuberculosis and respiratory diseases 2010;33(11):844-8

41. Lee CH, Liam CK, Pang YK, et al. Successful pregnancy with epidermal growth factor receptor tyrosine kinase inhibitor treatment of metastatic lung adenocarcinoma presenting with respiratory failure. Lung cancer (Amsterdam, Netherlands) 2011;74(2):349-51, doi:10.1016/j.lungcan.2011.08.008

42. Rivas G, Llinás N, Bonilla C, et al. Use of erlotinib throughout pregnancy: a case-report of a patient with metastatic lung adenocarcinoma. Lung cancer (Amsterdam, Netherlands) 2012;77(2):469-72, doi:10.1016/j.lungcan.2012.03.026

43. Boussios S, Han SN, Fruscio R, et al. Lung cancer in pregnancy: report of nine cases from an international collaborative study. Lung cancer (Amsterdam, Netherlands) 2013;82(3):499-505, doi:10.1016/j.lungcan.2013.09.002

44. Sarıman N, Levent E, Yener NA, et al. Lung cancer and pregnancy. Lung cancer (Amsterdam, Netherlands) 2013;79(3):321-3, doi:10.1016/j.lungcan.2012.11.014

45. Gil S, Goetgheluck J, Paci A, et al. Efficacy and safety of gefitinib during pregnancy: case report and literature review. Lung cancer (Amsterdam, Netherlands) 2014;85(3):481-4, doi:10.1016/j.lungcan.2014.06.003

46. Hayama M, Chida M, Tamura M, et al. Unexpected rapid growth of estrogen receptor positive lung cancer during pregnancy. Annals of thoracic and cardiovascular surgery : official journal of the Association of Thoracic and Cardiovascular Surgeons of Asia 2014;20(4):325-8, doi:10.5761/atcs.cr.12.01934

47. Kim JW, Kim JS, Cho JY, et al. Successful video-assisted thoracoscopic lobectomy in a pregnant woman with lung cancer. Lung cancer (Amsterdam, Netherlands) 2014;85(2):331-4, doi:10.1016/j.lungcan.2014.05.022

48. Neves I, Mota PC, Hespanhol VP. Lung cancer during pregnancy: an unusual case. Revista portuguesa de pneumologia 2014;20(1):46-9, doi:10.1016/j.rppneu.2013.06.005

49. Ceaușu M, Hostiuc S, Sajin M, et al. Gestational lung adenocarcinoma: case report. International journal of surgical pathology 2014;22(7):663-6, doi:10.1177/1066896914531816

50. Holzmann K, Kropfmüller R, Schinko H, et al. Lung cancer in pregnancy. Wiener klinische Wochenschrift 2015;127(15-16):639-44, doi:10.1007/s00508-015-0726-6

51. Ji Y, Schwartz J, Hartford A, et al. Successful Treatment of Non-Small Cell Lung Cancer With Erlotinib Throughout Pregnancy. JAMA oncology 2015;1(6):838-40, doi:10.1001/jamaoncol.2015.1300

52. Yates R, Zhang J. Lung Cancer in Pregnancy: An Unusual Case of Complete Response to Chemotherapy. Cureus 2015;7(12):e440, doi:10.7759/cureus.440

53. Cherian SV, Akasapu K, Kumar A, et al. A 34-Year-Old Pregnant Woman With Cough, Chest Pain, and a Left Upper Lobe Mass. Chest 2016;150(3):e87-91, doi:10.1016/j.chest.2016.03.008

54. Dagogo-Jack I, Gainor JF, Porter RL, et al. Clinicopathologic Features of NSCLC Diagnosed During Pregnancy or the Peripartum Period in the Era of Molecular Genotyping. Journal of thoracic oncology : official publication of the International Association for the Study of Lung Cancer 2016;11(9):1522-8, doi:10.1016/j.jtho.2016.05.031

55. Safini F, Jjouhadi H, Chehal A, et al. [Small cell bronchial carcinoma and pregnancy: about a case with review of the literature]. The Pan African medical journal 2016;23(130, doi:10.11604/pamj.2016.23.130.7856

56. Gziri MM, Brunée L, Cayphas C, et al. Low-grade bronchial mucoepidermoid carcinoma during pregnancy successfully treated by lobectomy. Journal of obstetrics and gynaecology : the journal of the Institute of Obstetrics and Gynaecology 2017;37(8):1082-1084, doi:10.1080/01443615.2017.1308325

57. Moro F, Mascilini F, Casella E, et al. Small cell lung cancer metastatic to the ovary diagnosed during pregnancy. Ultrasound in obstetrics & gynecology : the official journal of the International Society of Ultrasound in Obstetrics and Gynecology 2017;50(6):802-803, doi:10.1002/uog.17437

58. Isci Bostanci E, Guler I, Erdem O, et al. A rare case of ovarian cancer in a pregnant woman with metastatic non-small cell lung cancer. Journal of obstetrics and gynaecology : the journal of the Institute of Obstetrics and Gynaecology 2018;38(3):425-426, doi:10.1080/01443615.2017.1343292

59. Komura M, Yagishita S, Nakamura K, et al. A Case of a Pregnant Woman Diagnosed as Having ALK-rearranged Lung Adenocarcinoma. In vivo (Athens, Greece) 2018;32(5):1205-1209, doi:10.21873/invivo.11365

60. Padrão E, Melo C, Fernandes G, et al. Lung cancer in pregnancy - Report of a case treated with crizotinib. Pulmonology 2018;24(3):205-207, doi:10.1016/j.pulmoe.2018.03.007

61. Bellido C, Barbero P, Forcén L, et al. Lung adenocarcinoma during pregnancy: clinical case and literature review. The journal of maternal-fetal & neonatal medicine : the official journal of the European Association of Perinatal Medicine, the Federation of Asia and Oceania Perinatal Societies, the International Society of Perinatal Obstet 2019;32(19):3300-3302, doi:10.1080/14767058.2018.1461830

62. Jensen KH, Persson G, Storgaard L, et al. Antineoplastic treatment with crizotinib during pregnancy: a case report. Acta oncologica (Stockholm, Sweden) 2019;58(1):121-122, doi:10.1080/0284186x.2018.1497302

63. Siddiqui HU, Tang A, Raymond DP. Transformation of Recurrent Respiratory Papillomatosis: Squamous Cell Carcinoma in a Pregnant Teen. The Annals of thoracic surgery 2019;107(6):e403-e404, doi:10.1016/j.athoracsur.2018.10.047

64. Watanabe T, Yamashita T, Sugawara H, et al. Rapid Progression of Lung Cancer Following Emergency Caesarean Section Led to Postpartum Acute Respiratory Failure. Internal medicine (Tokyo, Japan) 2019;58(7):991-997, doi:10.2169/internalmedicine.1105-18

65. Han X, Yang H, Zhang Y, et al. Three cases of lung cancer in pregnancy and literature review. Annals of palliative medicine 2020;9(4):1928-1936, doi:10.21037/apm-20-999

66. Lin L, Sun J, Wang J. Lung cancer and intraocular metastasis in gestation: Clinical experiences of a rare case. Thoracic cancer 2020;11(9):2723-2726, doi:10.1111/1759-7714.13572

67. Boudy AS, Grausz N, Selleret L, et al. Use of tyrosine kinase inhibitors during pregnancy for oncogenic-driven advanced non-small cell lung carcinoma. Lung cancer (Amsterdam, Netherlands) 2021;161(68-75, doi:10.1016/j.lungcan.2021.09.001

68. Scarfone G, Fumagalli M, Imbimbo M, et al. First Case Report of Pregnancy on Alectinib in a Woman With Metastatic ALK-Rearranged Lung Cancer: A Case Report. Journal of thoracic oncology : official publication of the International Association for the Study of Lung Cancer 2021;16(5):873-877, doi:10.1016/j.jtho.2021.02.005
